# Ethyl 4-(4-bromo­phen­yl)-6-(4-ethoxy­phen­yl)-2-oxocyclo­hex-3-enecarboxyl­ate

**DOI:** 10.1107/S1600536809003523

**Published:** 2009-02-06

**Authors:** Amir Badshah, Aurangzeb Hasan, Cecilia R. Barbarín

**Affiliations:** a Department of Chemistry, Quaid-i-Azam University, Islamabad 45320, Pakistan; b División de Estudios de Posgrado, Facultad de Ciencias Químicas, UANL, Guerreo y Progreso S/N, Col. Treviño, CP, 64570 Monterrey, NL, Mexico

## Abstract

The title compound, C_23_H_23_BrO_4_, is an inter­mediate in the synthesis of fused heterocycles. In the title mol­ecule, the cyclo­hexene ring has a distorted half-chair conformation. The bromo­phenyl ring and the mean plane of the cyclo­hexene ring form a dihedral angle of 13.8 (3)°, whereas the benzene and cyclo­hexene rings are approximately perpendicular [88.44 (17)°]. There are only weak C—H⋯O and C—H⋯π inter­molecular inter­actions.

## Related literature

For applications of cyclo­hexenones, see: Eddington *et al.* (2000 ▶); Li & Strobel (2001 ▶); Luu *et al.* (2000 ▶); Padmavathi *et al.* (2000 ▶, 2001 ▶).
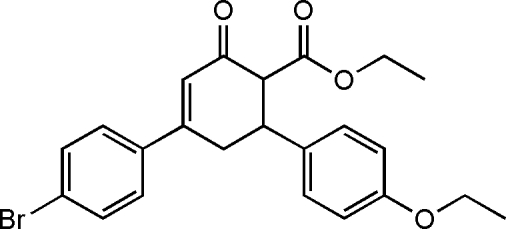


## Experimental

### 

#### Crystal data

C_23_H_23_BrO_4_
*M* *_r_* = 443.32
Monoclinic, 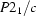

*a* = 12.792 (4) Å
*b* = 14.537 (4) Å
*c* = 12.114 (4) Å
β = 113.88 (2)°
*V* = 2059.8 (11) Å^3^
*Z* = 4
Mo *K*α radiation
μ = 2.02 mm^−1^
*T* = 298 (2) K
0.50 × 0.50 × 0.08 mm

#### Data collection

Bruker P4 diffractometer
Absorption correction: gaussian (*XSCANS*; Bruker, 1999 ▶) *T*_min_ = 0.246, *T*_max_ = 0.941
7765 measured reflections
3630 independent reflections
2088 reflections with *I* > 2σ(*I*)
*R*_int_ = 0.054
3 standard reflections every 97 reflections intensity decay: 6.4%

#### Refinement

*R*[*F* ^2^ > 2σ(*F* ^2^)] = 0.059
*wR*(*F* ^2^) = 0.175
*S* = 1.01
3630 reflections
254 parameters
H-atom parameters constrained
Δρ_max_ = 0.44 e Å^−3^
Δρ_min_ = −0.41 e Å^−3^

### 

Data collection: *XSCANS* (Bruker, 1999 ▶); cell refinement: *XSCANS*; data reduction: *XSCANS*; program(s) used to solve structure: *SHELXTL-Plus* (Sheldrick, 2008 ▶); program(s) used to refine structure: *SHELXTL-Plus*; molecular graphics: *SHELXTL-Plus* and *Mercury* (Macrae *et al.*, 2006 ▶); software used to prepare material for publication: *SHELXTL-Plus*.

## Supplementary Material

Crystal structure: contains datablocks I, global. DOI: 10.1107/S1600536809003523/gk2184sup1.cif

Structure factors: contains datablocks I. DOI: 10.1107/S1600536809003523/gk2184Isup2.hkl

Additional supplementary materials:  crystallographic information; 3D view; checkCIF report

## Figures and Tables

**Table 1:** Hydrogen-bond geometry (Å, °)

*D*—H⋯*A*	*D*—H	H⋯*A*	*D*⋯*A*	*D*—H⋯*A*
C5—H5*A*⋯O1^i^	0.93	2.42	3.163 (6)	137
C8—H8*A*⋯O2^ii^	0.97	2.59	3.244 (6)	125
C15—H15*B*⋯O2	0.96	2.58	3.062 (13)	111
C23—H23*A*⋯*Cg*^iii^	0.96	2.90	3.741 (6)	147

Symmetry codes: (i) 
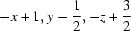
; (ii) 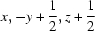; (iii) 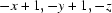.

**Fig. 1.**
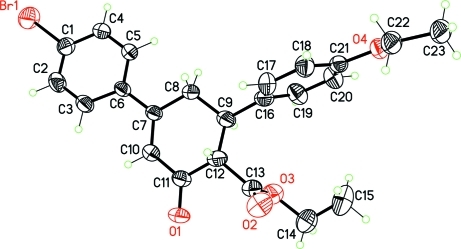
The title molecule with the atom labeling scheme. The displacement ellipsoids are drawn at the 30% probability level and H atoms are shown as small spheres of arbitrary radii.

**Fig. 2.**
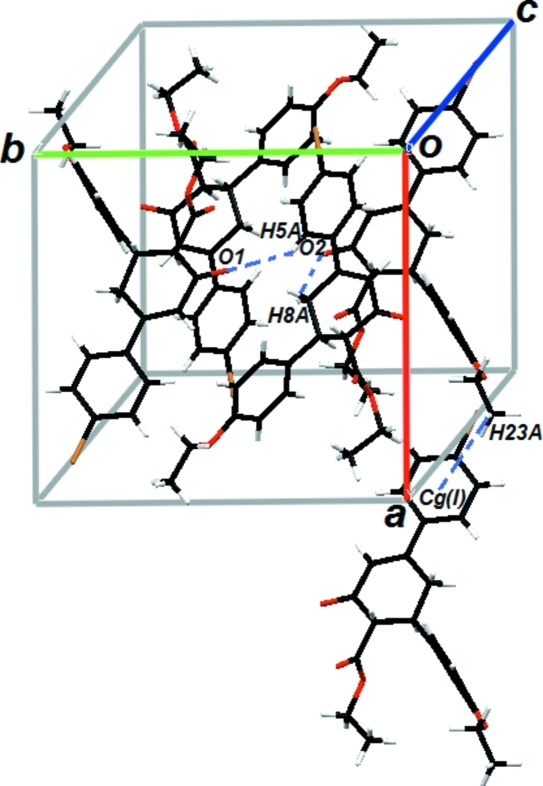
The packing diagram of the title compound, viewed along the c axis showing weak C—H···O and C—H···π interactions

## supplementary crystallographic information

### Comment

Cyclohexenones are either prepared from natural sources or entirely *via* synthetic routes. The reason for their preparation is a variety of medical effects. The molecules provide anticonvulsant, antimalarial, antiinflamatory and cardiovascular effects (Eddington *et al.*, 2000). Cyclohexenones are also important intermediates for many biologically active compounds (Padmavathi *et al.*, 2001; Padmavathi *et al.*, 2000). A series of novel compounds have been synthesized, known as cyclohexenoic long chain fatty alcohols, which are used in the treatment of neurological disorders (Luu *et al.*, 2000). A number of their derivatives have fungicidal and antitumor activities (Li & Strobel, 2001).

In the title compound, C_23_H_23_BrO_4_ (Scheme 1, Fig. 1), the two rings, *i.e.* bromophenyl [C1-C6] and the cyclohexene [C7-C12], are slightly twisted' with the dihedral angle of 13.8 (3)°. Cyclohexene [C7- C12], is approximately perpendicular to the benzene ring [C16-C21] [88.44 (17)°]. The title molecule has two asymmetric carbon atoms C9 and C12 that are in *RS* and *SR* configurations, respectively. The comformation of the cyclohexene ring is distorted half chair [Θ = 50.6 (10) and Φ = 138.9 (13)°, compared with the ideal values of Θ = 50.0 and Φ = 150.0°].

As indicated by intermolecular contacts. there are only weak intermolecular interactions *X*—H···O and C—H···π (Table 1). The crystal packing is shown in Fig. 2.

### Experimental

Ethyl 4-(4-bromophenyl)-6-(4-ethoxyphenyl)-2-oxocyclhex-3-enecarboxylate was synthesized by refluxing ethyl acetoacetate (0.39 g, 0.40 ml, 3 mmol) with 1-(4-bromophenyl)-3-(4-ethoxyphenyl) prop-2-ene-1-one (3 mmol, 0.990 g) for 2 h in 10–15 ml of ethanol in presence of 0.5 ml 10% NaOH. The reaction mixture was then poured while having been stirred intensively into 200 ml of ice-cold water. The mixture was kept at room temperature until the reaction product separated as a solid, which was filtered off and recrystallized from ethanol (yield 65%, m.p. 400 K).

### Refinement

All the hydrogen atoms have been found in a difference Fourier map, nevertheless, they were placed in idealized positions and refined as riding atoms at constrained distances: aromatic C—H = 0.93, C_methylene_—H=0.97, C_methine_—H=0.98 and methyl C—H = 0.96 Å, while *U*_iso_H=1.5*U*_eq_C_methyl_ or 1.2*U*_eq_C_aryl/methylene/methine_.

### Crystal data

**Table:**

C_23_H_23_BrO_4_	*F*(000) = 912
*M**_r_* = 443.32	*D*_x_ = 1.430 Mg m^−^^3^
Monoclinic, *P*2_1_/*c*	Melting point: 400 K
Hall symbol: -P 2ybc	Mo *K*α radiation, λ = 0.71073 Å
*a* = 12.792 (4) Å	Cell parameters from 91 reflections
*b* = 14.537 (4) Å	θ = 4.6–12.4°
*c* = 12.114 (4) Å	µ = 2.02 mm^−^^1^
β = 113.88 (2)°	*T* = 298 K
*V* = 2059.8 (11) Å^3^	Plate, colourless
*Z* = 4	0.50 × 0.50 × 0.08 mm

### Data collection

**Table:**

Bruker P4 diffractometer	2088 reflections with *I* > 2σ(*I*)
Radiation source: fine-focus sealed tube	*R*_int_ = 0.054
graphite	θ_max_ = 25.0°, θ_min_ = 2.2°
ω scans	*h* = −14→15
Absorption correction: gaussian (*XSCANS*; Bruker, 1999)	*k* = −1→17
*T*_min_ = 0.246, *T*_max_ = 0.941	*l* = −14→14
7765 measured reflections	3 standard reflections every 97 reflections
3630 independent reflections	intensity decay: 6.4%

### Refinement

**Table:**

Refinement on *F*^2^	Secondary atom site location: difference Fourier map
Least-squares matrix: full	Hydrogen site location: inferred from neighbouring sites
*R*[*F*^2^ > 2σ(*F*^2^)] = 0.059	H-atom parameters constrained
*wR*(*F*^2^) = 0.175	*w* = 1/[σ^2^(*F*_o_^2^) + (0.0744*P*)^2^ + 1.4731*P*] where *P* = (*F*_o_^2^ + 2*F*_c_^2^)/3
*S* = 1.01	(Δ/σ)_max_ < 0.001
3630 reflections	Δρ_max_ = 0.44 e Å^−^^3^
254 parameters	Δρ_min_ = −0.41 e Å^−^^3^
0 restraints	Extinction correction: *SHELXTL-Plus* (Sheldrick, 2008), Fc^*^=kFc[1+0.001xFc^2^λ^3^/sin(2θ)]^-1/4^
Primary atom site location: structure-invariant direct methods	Extinction coefficient: 0.0050 (10)

### Special details

**Table:**

Experimental. Absorption correction based on 6 crystal faces Faces used: 001, 00–1, 20–1, -201, 010, 0–10
Geometry. All e.s.d.'s (except the e.s.d. in the dihedral angle between two l.s. planes) are estimated using the full covariance matrix. The cell e.s.d.'s are taken into account individually in the estimation of e.s.d.'s in distances, angles and torsion angles; correlations between e.s.d.'s in cell parameters are only used when they are defined by crystal symmetry. An approximate (isotropic) treatment of cell e.s.d.'s is used for estimating e.s.d.'s involving l.s. planes.
Refinement. Refinement of *F*^2^ against ALL reflections. The weighted *R*-factor *wR* and goodness of fit *S* are based on *F*^2^, conventional *R*-factors *R* are based on *F*, with *F* set to zero for negative *F*^2^. The threshold expression of *F*^2^ > σ(*F*^2^) is used only for calculating *R*-factors(gt) *etc*. and is not relevant to the choice of reflections for refinement. *R*-factors based on *F*^2^ are statistically about twice as large as those based on *F*, and *R*- factors based on ALL data will be even larger.

### Fractional atomic coordinates and isotropic or equivalent isotropic displacement parameters (Å^2^)

**Table:**

	*x*	*y*	*z*	*U*_iso_*/*U*_eq_	
Br1	0.12714 (6)	0.10565 (5)	1.01969 (7)	0.1061 (4)	
O1	0.5590 (3)	0.4373 (2)	0.7177 (4)	0.0864 (11)	
O2	0.6752 (4)	0.3470 (3)	0.5468 (4)	0.1049 (13)	
O3	0.7863 (4)	0.3351 (3)	0.7398 (4)	0.0901 (11)	
O4	0.8559 (3)	−0.0461 (2)	0.5587 (3)	0.0662 (9)	
C1	0.2279 (4)	0.1456 (4)	0.9511 (5)	0.0673 (13)	
C2	0.2177 (5)	0.2318 (4)	0.9038 (5)	0.0777 (15)	
H2A	0.1619	0.2715	0.9068	0.093*	
C3	0.2889 (4)	0.2597 (3)	0.8525 (5)	0.0700 (13)	
H3A	0.2809	0.3185	0.8200	0.084*	
C4	0.3099 (4)	0.0866 (3)	0.9484 (5)	0.0687 (13)	
H4A	0.3174	0.0281	0.9818	0.082*	
C5	0.3812 (4)	0.1153 (3)	0.8955 (5)	0.0635 (12)	
H5A	0.4362	0.0749	0.8922	0.076*	
C6	0.3739 (4)	0.2020 (3)	0.8474 (4)	0.0534 (11)	
C7	0.4524 (4)	0.2326 (3)	0.7936 (4)	0.0523 (10)	
C8	0.5179 (4)	0.1615 (3)	0.7592 (4)	0.0591 (12)	
H8A	0.5465	0.1162	0.8234	0.071*	
H8B	0.4660	0.1302	0.6871	0.071*	
C9	0.6161 (5)	0.1980 (3)	0.7360 (6)	0.0819 (16)	
H9A	0.6733	0.2120	0.8171	0.098*	
C10	0.4667 (4)	0.3220 (3)	0.7762 (5)	0.0649 (13)	
H10A	0.4249	0.3647	0.7988	0.078*	
C11	0.5422 (4)	0.3557 (3)	0.7250 (5)	0.0664 (13)	
C12	0.5970 (5)	0.2855 (3)	0.6760 (6)	0.0923 (19)	
H12A	0.5368	0.2717	0.5965	0.111*	
C13	0.6893 (5)	0.3258 (3)	0.6454 (7)	0.0745 (15)	
C14	0.8792 (5)	0.3710 (5)	0.7141 (8)	0.122 (3)	
H14A	0.9372	0.3963	0.7873	0.147*	
H14B	0.8510	0.4203	0.6554	0.147*	
C15	0.9299 (8)	0.2991 (7)	0.6667 (11)	0.176 (4)	
H15A	0.9915	0.3247	0.6506	0.264*	
H15B	0.8728	0.2748	0.5934	0.264*	
H15C	0.9587	0.2506	0.7251	0.264*	
C16	0.6757 (5)	0.1277 (3)	0.6896 (5)	0.0666 (13)	
C17	0.6243 (4)	0.0920 (4)	0.5765 (6)	0.0792 (15)	
H17A	0.5488	0.1082	0.5298	0.095*	
C18	0.6789 (4)	0.0329 (4)	0.5277 (5)	0.0737 (14)	
H18A	0.6411	0.0099	0.4499	0.088*	
C19	0.7853 (5)	0.0997 (3)	0.7578 (5)	0.0791 (15)	
H19A	0.8221	0.1205	0.8368	0.095*	
C20	0.8414 (5)	0.0416 (4)	0.7115 (5)	0.0747 (14)	
H20A	0.9160	0.0240	0.7593	0.090*	
C21	0.7896 (4)	0.0092 (3)	0.5964 (4)	0.0574 (11)	
C22	0.8162 (5)	−0.0624 (4)	0.4328 (5)	0.0775 (14)	
H22A	0.7985	−0.0046	0.3891	0.093*	
H22B	0.7475	−0.0997	0.4051	0.093*	
C23	0.9080 (5)	−0.1110 (4)	0.4114 (6)	0.0858 (17)	
H23A	0.8839	−0.1220	0.3265	0.129*	
H23B	0.9237	−0.1687	0.4536	0.129*	
H23C	0.9759	−0.0739	0.4402	0.129*	

### Atomic displacement parameters (Å^2^)

**Table:**

	*U*^11^	*U*^22^	*U*^33^	*U*^12^	*U*^13^	*U*^23^
Br1	0.1224 (6)	0.1085 (6)	0.1275 (7)	−0.0075 (4)	0.0918 (5)	−0.0037 (4)
O1	0.107 (3)	0.0405 (19)	0.126 (3)	−0.0019 (18)	0.061 (2)	0.0037 (19)
O2	0.105 (3)	0.111 (3)	0.100 (3)	−0.012 (3)	0.042 (3)	0.002 (3)
O3	0.085 (3)	0.084 (3)	0.100 (3)	−0.009 (2)	0.036 (3)	0.000 (2)
O4	0.068 (2)	0.065 (2)	0.072 (2)	0.0053 (16)	0.0340 (18)	−0.0070 (17)
C1	0.076 (3)	0.067 (3)	0.072 (3)	−0.006 (3)	0.043 (3)	−0.012 (3)
C2	0.085 (4)	0.072 (4)	0.093 (4)	0.016 (3)	0.054 (3)	−0.005 (3)
C3	0.080 (3)	0.054 (3)	0.086 (4)	0.012 (2)	0.044 (3)	0.003 (2)
C4	0.075 (3)	0.051 (3)	0.086 (4)	0.000 (2)	0.039 (3)	0.001 (2)
C5	0.062 (3)	0.051 (3)	0.084 (4)	0.002 (2)	0.037 (3)	−0.005 (2)
C6	0.060 (3)	0.041 (2)	0.061 (3)	0.004 (2)	0.025 (2)	−0.005 (2)
C7	0.057 (3)	0.041 (2)	0.058 (3)	0.0010 (19)	0.022 (2)	−0.003 (2)
C8	0.065 (3)	0.042 (2)	0.078 (3)	−0.005 (2)	0.037 (3)	−0.003 (2)
C9	0.106 (4)	0.048 (3)	0.124 (5)	0.007 (3)	0.080 (4)	0.005 (3)
C10	0.067 (3)	0.050 (3)	0.083 (4)	0.003 (2)	0.036 (3)	−0.004 (2)
C11	0.071 (3)	0.047 (3)	0.079 (4)	0.000 (2)	0.028 (3)	0.000 (2)
C12	0.111 (4)	0.049 (3)	0.153 (6)	−0.006 (3)	0.091 (4)	0.003 (3)
C13	0.081 (4)	0.052 (3)	0.101 (5)	−0.010 (3)	0.048 (4)	−0.006 (3)
C14	0.065 (4)	0.109 (5)	0.189 (8)	−0.015 (4)	0.046 (5)	−0.004 (5)
C15	0.128 (7)	0.185 (9)	0.266 (12)	0.007 (7)	0.133 (8)	0.023 (9)
C16	0.089 (4)	0.047 (3)	0.082 (4)	−0.006 (3)	0.053 (3)	−0.001 (3)
C17	0.057 (3)	0.084 (4)	0.099 (4)	0.010 (3)	0.033 (3)	0.003 (3)
C18	0.061 (3)	0.080 (3)	0.079 (4)	−0.001 (3)	0.027 (3)	−0.014 (3)
C19	0.095 (4)	0.074 (3)	0.072 (4)	0.018 (3)	0.038 (3)	0.002 (3)
C20	0.073 (3)	0.077 (3)	0.068 (4)	0.016 (3)	0.022 (3)	−0.004 (3)
C21	0.068 (3)	0.049 (2)	0.063 (3)	−0.002 (2)	0.034 (3)	0.001 (2)
C22	0.081 (3)	0.081 (3)	0.079 (4)	−0.006 (3)	0.041 (3)	−0.016 (3)
C23	0.094 (4)	0.090 (4)	0.091 (4)	−0.013 (3)	0.056 (3)	−0.027 (3)

### Geometric parameters (Å, °)

**Table:**

Br1—C1	1.886 (5)	C10—H10A	0.9300
O1—C11	1.216 (6)	C11—C12	1.490 (7)
O2—C13	1.176 (7)	C12—C13	1.493 (7)
O3—C13	1.311 (7)	C12—H12A	0.9800
O3—C14	1.442 (7)	C14—C15	1.465 (11)
O4—C21	1.374 (5)	C14—H14A	0.9700
O4—C22	1.419 (6)	C14—H14B	0.9700
C1—C2	1.361 (7)	C15—H15A	0.9600
C1—C4	1.366 (7)	C15—H15B	0.9600
C2—C3	1.357 (7)	C15—H15C	0.9600
C2—H2A	0.9300	C16—C17	1.360 (8)
C3—C6	1.393 (6)	C16—C19	1.370 (7)
C3—H3A	0.9300	C17—C18	1.382 (7)
C4—C5	1.375 (7)	C17—H17A	0.9300
C4—H4A	0.9300	C18—C21	1.365 (7)
C5—C6	1.375 (6)	C18—H18A	0.9300
C5—H5A	0.9300	C19—C20	1.366 (7)
C6—C7	1.470 (6)	C19—H19A	0.9300
C7—C10	1.342 (6)	C20—C21	1.363 (7)
C7—C8	1.492 (6)	C20—H20A	0.9300
C8—C9	1.492 (6)	C22—C23	1.480 (7)
C8—H8A	0.9700	C22—H22A	0.9700
C8—H8B	0.9700	C22—H22B	0.9700
C9—C12	1.436 (7)	C23—H23A	0.9600
C9—C16	1.512 (6)	C23—H23B	0.9600
C9—H9A	0.9800	C23—H23C	0.9600
C10—C11	1.429 (7)		
			
C13—O3—C14	114.9 (5)	C13—C12—H12A	102.8
C21—O4—C22	117.1 (4)	O2—C13—O3	124.1 (5)
C2—C1—C4	120.8 (5)	O2—C13—C12	123.0 (7)
C2—C1—Br1	120.2 (4)	O3—C13—C12	112.9 (6)
C4—C1—Br1	119.1 (4)	O3—C14—C15	111.2 (6)
C3—C2—C1	120.0 (5)	O3—C14—H14A	109.4
C3—C2—H2A	120.0	C15—C14—H14A	109.4
C1—C2—H2A	120.0	O3—C14—H14B	109.4
C2—C3—C6	121.4 (5)	C15—C14—H14B	109.4
C2—C3—H3A	119.3	H14A—C14—H14B	108.0
C6—C3—H3A	119.3	C14—C15—H15A	109.5
C1—C4—C5	118.8 (5)	C14—C15—H15B	109.5
C1—C4—H4A	120.6	H15A—C15—H15B	109.5
C5—C4—H4A	120.6	C14—C15—H15C	109.5
C4—C5—C6	122.1 (4)	H15A—C15—H15C	109.5
C4—C5—H5A	119.0	H15B—C15—H15C	109.5
C6—C5—H5A	119.0	C17—C16—C19	116.9 (5)
C5—C6—C3	117.0 (4)	C17—C16—C9	121.4 (5)
C5—C6—C7	121.5 (4)	C19—C16—C9	121.6 (5)
C3—C6—C7	121.5 (4)	C16—C17—C18	123.1 (5)
C10—C7—C6	121.6 (4)	C16—C17—H17A	118.4
C10—C7—C8	120.0 (4)	C18—C17—H17A	118.4
C6—C7—C8	118.5 (4)	C21—C18—C17	118.4 (5)
C9—C8—C7	114.7 (4)	C21—C18—H18A	120.8
C9—C8—H8A	108.6	C17—C18—H18A	120.8
C7—C8—H8A	108.6	C20—C19—C16	121.1 (5)
C9—C8—H8B	108.6	C20—C19—H19A	119.5
C7—C8—H8B	108.6	C16—C19—H19A	119.5
H8A—C8—H8B	107.6	C21—C20—C19	121.0 (5)
C12—C9—C8	115.2 (4)	C21—C20—H20A	119.5
C12—C9—C16	114.7 (4)	C19—C20—H20A	119.5
C8—C9—C16	114.8 (4)	C20—C21—C18	119.4 (4)
C12—C9—H9A	103.2	C20—C21—O4	115.7 (4)
C8—C9—H9A	103.2	C18—C21—O4	124.9 (4)
C16—C9—H9A	103.2	O4—C22—C23	107.7 (4)
C7—C10—C11	124.0 (4)	O4—C22—H22A	110.2
C7—C10—H10A	118.0	C23—C22—H22A	110.2
C11—C10—H10A	118.0	O4—C22—H22B	110.2
O1—C11—C10	122.4 (5)	C23—C22—H22B	110.2
O1—C11—C12	121.0 (4)	H22A—C22—H22B	108.5
C10—C11—C12	116.6 (4)	C22—C23—H23A	109.5
C9—C12—C11	114.6 (5)	C22—C23—H23B	109.5
C9—C12—C13	118.9 (5)	H23A—C23—H23B	109.5
C11—C12—C13	112.1 (4)	C22—C23—H23C	109.5
C9—C12—H12A	102.8	H23A—C23—H23C	109.5
C11—C12—H12A	102.8	H23B—C23—H23C	109.5
			
C4—C1—C2—C3	0.5 (9)	C10—C11—C12—C9	−30.2 (8)
Br1—C1—C2—C3	−178.7 (4)	O1—C11—C12—C13	12.5 (8)
C1—C2—C3—C6	−0.4 (9)	C10—C11—C12—C13	−169.7 (5)
C2—C1—C4—C5	−0.9 (8)	C14—O3—C13—O2	−2.3 (8)
Br1—C1—C4—C5	178.3 (4)	C14—O3—C13—C12	178.0 (5)
C1—C4—C5—C6	1.2 (8)	C9—C12—C13—O2	123.8 (7)
C4—C5—C6—C3	−1.2 (7)	C11—C12—C13—O2	−98.6 (7)
C4—C5—C6—C7	178.6 (4)	C9—C12—C13—O3	−56.5 (7)
C2—C3—C6—C5	0.7 (8)	C11—C12—C13—O3	81.1 (6)
C2—C3—C6—C7	−179.0 (5)	C13—O3—C14—C15	−79.5 (8)
C5—C6—C7—C10	−162.0 (5)	C12—C9—C16—C17	−66.9 (7)
C3—C6—C7—C10	17.7 (7)	C8—C9—C16—C17	70.0 (7)
C5—C6—C7—C8	17.5 (6)	C12—C9—C16—C19	110.6 (6)
C3—C6—C7—C8	−162.8 (5)	C8—C9—C16—C19	−112.4 (6)
C10—C7—C8—C9	14.6 (7)	C19—C16—C17—C18	−2.6 (8)
C6—C7—C8—C9	−164.8 (4)	C9—C16—C17—C18	175.1 (5)
C7—C8—C9—C12	−37.7 (7)	C16—C17—C18—C21	0.1 (8)
C7—C8—C9—C16	−174.4 (5)	C17—C16—C19—C20	2.8 (8)
C6—C7—C10—C11	179.6 (4)	C9—C16—C19—C20	−174.8 (5)
C8—C7—C10—C11	0.2 (8)	C16—C19—C20—C21	−0.6 (8)
C7—C10—C11—O1	−175.0 (5)	C19—C20—C21—C18	−2.0 (8)
C7—C10—C11—C12	7.3 (8)	C19—C20—C21—O4	177.9 (4)
C8—C9—C12—C11	45.5 (8)	C17—C18—C21—C20	2.2 (7)
C16—C9—C12—C11	−177.8 (5)	C17—C18—C21—O4	−177.6 (4)
C8—C9—C12—C13	−178.0 (5)	C22—O4—C21—C20	−164.8 (4)
C16—C9—C12—C13	−41.2 (9)	C22—O4—C21—C18	15.0 (6)
O1—C11—C12—C9	152.0 (6)	C21—O4—C22—C23	171.5 (4)

### Hydrogen-bond geometry (Å, °)

**Table:**

*D*—H···*A*	*D*—H	H···*A*	*D*···*A*	*D*—H···*A*
C5—H5A···O1^i^	0.93	2.42	3.163 (6)	137
C8—H8A···O2^ii^	0.97	2.59	3.244 (6)	125
C15—H15B···O2	0.96	2.58	3.062 (13)	111
C23—H23A···Cg^iii^	0.96	2.90	3.741 (6)	147

Symmetry codes: (i) −*x*+1, *y*−1/2, −*z*+3/2; (ii) *x*, −*y*+1/2, *z*+1/2;  (iii) −*x*+1, −*y*+1, −*z*.
